# Psychotic disorders and splenium of the corpus callosum tumor: diagnostic and therapeutic approach

**DOI:** 10.1192/j.eurpsy.2025.2253

**Published:** 2025-08-26

**Authors:** S. Ben Aissa, K. Razki, C. Najar, A. Larnaout, W. Melki

**Affiliations:** 1 Psychiatry, Razi hospital, Manouba, Tunisia

## Abstract

**Introduction:**

Psychosis encompasses a complex spectrum of psychiatric manifestations that can arise from diverse organic etiologies, such as severe head trauma (1), epilepsy, systemic diseases, and tumors. While brain tumors are typically considered neurological conditions primarily affecting cognitive and motor functions, they can also present with psychiatric symptoms that exhibit clinical and evolutionary atypicality. This interplay between organic pathologies and psychiatric symptomatology underscores the intricate nature of mental health disorders originating from physiological disruptions.

**Objectives:**

To describe a case of chronic psychotic disorder with intermittent exacerbations secondary to a neurological tumor and discuss the diagnostic and therapeutic challenges.

**Methods:**

We report the case of a 46-year-old male patient who was admitted to Razi Hospital for aggressive behavior, delusional syndrome, and auditory hallucinations in 2023. The case report is followed by a discussion reviewing relevant literature on the subject.

**Results:**

A 46-year-old Caucasian man with no significant medical history was referred to our psychiatry service due to recent behavioral disturbances marked by hetero-aggressive outbursts. During evaluation, the patient was conscious, well-oriented in time and space, but anxious and attentive. He reported auditory hallucinations with religious content, persecutory delusions, and mystical-religious ideas. According to the patient’s history, auditory hallucinations began at age 16, with exacerbations at 25, 34, and 42 years.

Psychometric evaluation using the Positive and Negative Syndrome Scale (PANSS) yielded scores of 39 for positive symptoms and 16 for negative symptoms. Neurological examination revealed no abnormalities. Brain MRI showed a tumor in the splenium of the corpus callosum measuring 18 x 14 x 8 mm, with mild hypointensity on T1, hyperintensity on T2 and FLAIR, and minimal contrast enhancement suggesting a glial origin.

The patient was treated with risperidone (4 mg/day) and diazepam (5 mg/day). Over six weeks, PANSS scores decreased (positive: 23 points, negative: 7 points).

After consultation with the neurosurgery team, surgical intervention was not pursued due to the absence of tumor growth and neurological symptoms.

**Conclusions:**

The observed partial remission of psychotic symptoms with pharmacological intervention highlights the potential efficacy of such treatments in ameliorating psychiatric manifestations of organic brain pathologies.

Reference

(1) Khouloud Razki, Uta Ouali, Sofiene Ben Aissa, Chaima Najar, Amina Aissa, Raba Jomli, Journal of the Neurological Sciences, Volume 455, Supplement, December 2023, 122512, ISSN 0022-510X DOI : https://doi.org/10.1016/j.jns.2023.122512

**Disclosure of Interest:**

None Declared

